# The use of droplet-based microfluidic technologies for accelerated selection of *Yarrowia lipolytica* and *Phaffia rhodozyma* yeast mutants

**DOI:** 10.1093/biomethods/bpae049

**Published:** 2024-07-10

**Authors:** Taras Mika, Martins Kalnins, Kriss Spalvins

**Affiliations:** Institute of Energy Systems and Environment, Riga Technical University, 12 – K1 Āzene street, Riga, LV-1048, Latvia; Institute of Energy Systems and Environment, Riga Technical University, 12 – K1 Āzene street, Riga, LV-1048, Latvia; Institute of Energy Systems and Environment, Riga Technical University, 12 – K1 Āzene street, Riga, LV-1048, Latvia

**Keywords:** microfluidics, microorganisms, mutagenesis, microdroplet, selection

## Abstract

Microorganisms are widely used for the industrial production of various valuable products, such as pharmaceuticals, food and beverages, biofuels, enzymes, amino acids, vaccines, etc. Research is constantly carried out to improve their properties, mainly to increase their productivity and efficiency and reduce the cost of the processes. The selection of microorganisms with improved qualities takes a lot of time and resources (both human and material); therefore, this process itself needs optimization. In the last two decades, microfluidics technology appeared in bioengineering, which allows for manipulating small particles (from tens of microns to nanometre scale) in the flow of liquid in microchannels. The technology is based on small-volume objects (microdroplets from nano to femtolitres), which are manipulated using a microchip. The chip is made of an optically transparent inert to liquid medium material and contains a series of channels of small size (<1 mm) of certain geometry. Based on the physical and chemical properties of microparticles (like size, weight, optical density, dielectric constant, etc.), they are separated using microsensors. The idea of accelerated selection of microorganisms is the application of microfluidic technologies to separate mutants with improved qualities after mutagenesis. This article discusses the possible application and practical implementation of microfluidic separation of mutants, including yeasts like *Yarrowia lipolytica* and *Phaffia rhodozyma* after chemical mutagenesis will be discussed.

## Graphical Abstract

**Graphical Abstract:**
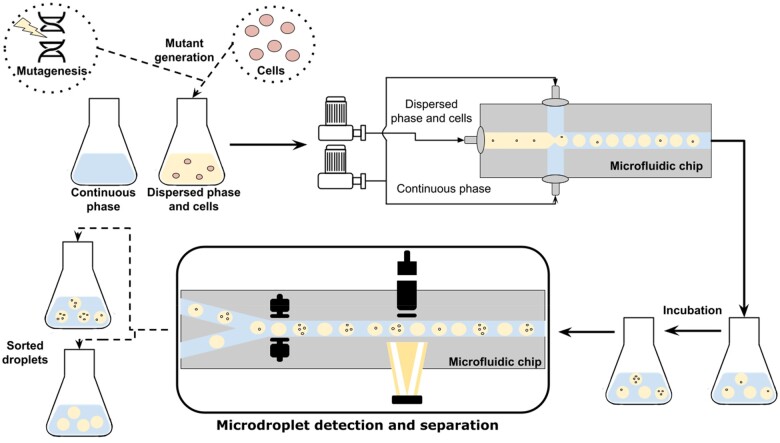

## Introduction

Yeast has many diverse applications, including the food industry, such as the production of beer and wine; the medical industry, such as food supplement production or the production of recombinant biopharmaceuticals; the biofuel industry, such as bioethanol and biobutanol production; and finally, the single-cell protein industry for the production of dietary protein and animal feed [1]. While yeast has been used for centuries, the selection and improvement of strains are still needed as the desired characteristics and requirements are constantly changing, along with the industry. Desired characteristics include productivity, range of substrate that can be utilized, substrate consumption efficiency, etc. Another strain improvement route is modifying metabolism and new substance production, such as human insulin. *Saccharomyces cerevisiae* is one of the most used strains in biotechnology, commonly used for beer, wine, and bread production, while strains such as *Scheffersomyces stipitis*, *Yarrowia lipolytica*, *Kluyveromyces lactis*, *Dekkera bruxellensis*, and *Phaffia rhodozyma* fall in nonconventional category [2].

*Phaffia rhodozyma* is a basidiomycetous yeast well-known for producing the carotenoid pigment astaxanthin. This pigment has many applications due to its bioactive and pigmentation properties. It is used as an additive in food and beverages, feed, nutraceuticals, pharmaceuticals, and cosmetics products [3–6]. Astaxanthin is an antioxidant used as a health supplement for humans [7]. *Phaffia rhodozyma* can produce substantial amounts of astaxanthin from industrial by-products, which is an attractive and feasible approach to meet the market needs of astaxanthin [8]. The oleaginous, strictly aerobic yeast *Y. lipolytica* represents a potential microbial cell factory for the recombinant production of various valuable products. The yeasts’ potential as a synthesizer of several high-value food ingredients, such as organic acids, aromas, and emulsifiers from a range of diverse substrates, from ethanol to olive oil waste, is of interest in a biorefinery context. *Yarrowia lipolytica* synthesizes a wide range of functional and bioactive compounds that can act as active ingredients in functional beverages [9–12].

Even though both microorganism species have beneficial industrial characteristics, they still need to be improved as their commercialization efforts require further development. To improve commercial suitability, mutants are often created and screened to find an enhancement, for example, in secondary metabolite production, biomass yield, growth speed, or stress tolerance. Mutants can be created using various methods through mutagenesis, but unfortunately, organism mutation rates are low. Thus, the selection requires many lines grown in parallel. Generally, this process requires a lot of human resources and time.

A technology called microfluidics is increasingly used in bioengineering. It allows to manipulate small-sized particles from tens of microns to nanometre scale such as droplets and cells. Based on the physical and chemical differences of particles, including size, weight, optical density, dielectric constant, etc., they can be recognized and separated from each other. Consequently, this technique is often used in screening mutants as it allows us to evaluate each mutant separately, which is not possible in flask experiments where the population of mutants is evaluated.

One of the first microdroplet cell encapsulations was published in 1945 by Joshua Lederberg [13]. From this first laborious, time-consuming, and low-throughput demonstration, microfluidic technology has evolved tremendously. It is constantly improving, and new prospects have appeared: DNA amplification, enzymatic assays, biochemical assays, nanoparticle research, medical diagnosis, and fundamental research [14–17]. Microfluidic technologies go far beyond the biotechnology boundaries [18–20]. However, this article will touch on a small field of microdroplet applications and focus on droplet-based microfluidic technologies for cell cultivation and sorting.

The goal of this review is to research possible concept solutions for the mutant selection of *P. rhodozyma* and *Y. lipolytica* using microdroplet technology by analysing already existing data from publications. We will try to find and discuss factors that may help to reveal previously not published solutions to create a stable, cost-effective, suitable setup for cell cultivation, test and sort mutants with high growth density and productivity. Also, the required hardware and software will be partially mentioned in this review. We will refer to publications outlining the basic principles of technology and the latest progress in the field when discussing a specific topic. This review is also intended as a guide for possibilities and considerations that must be assessed if one plans to use microdroplet technology for the selection of yeast mutants.

## Microorganisms, mutagenesis, and microfluidics in the accelerated selection of mutants

Both *P. rhodozyma* and *Y. lipolytica* have been extensively researched for their industrially valuable qualities, and both have been improved via mutagenesis and mutant screening [21–26]. Random mutagenesis is one of the most widely used methods for improving wild-type strains for industrial application as it does not depend on the host genetics, allowing it to be used for a variety of microorganisms. Another benefit is the obtained mutant library with high genetic diversity and, finally, the process does not involve direct genetic, modification which simplifies the certification process [27]. Considerations outlined in this article can also be appropriated for other species of yeast and other groups of microorganisms, but to narrow down the possibilities and to derive more specific conclusions for the selected technological solutions, we will focus on these two yeast species.

Mutants can be created using various methods, such as random mutagenesis using chemical mutagens, UV-radiation treatment, or biotechnological means, including gene editing. Chemical- and *UV*-mediated mutagenesis methods are one of the oldest and most widely used for their ease of use and low cost [28–32], while atmospheric and room temperature plasma-intermediated mutagenesis is one of the newest developments in the field [27]. To narrow down the focus of the article in further descriptions, we will refer to a well-known and still widely utilized ethyl methane sulfonate (EMS) chemical mutagenesis [33–35].

**Figure 1.**
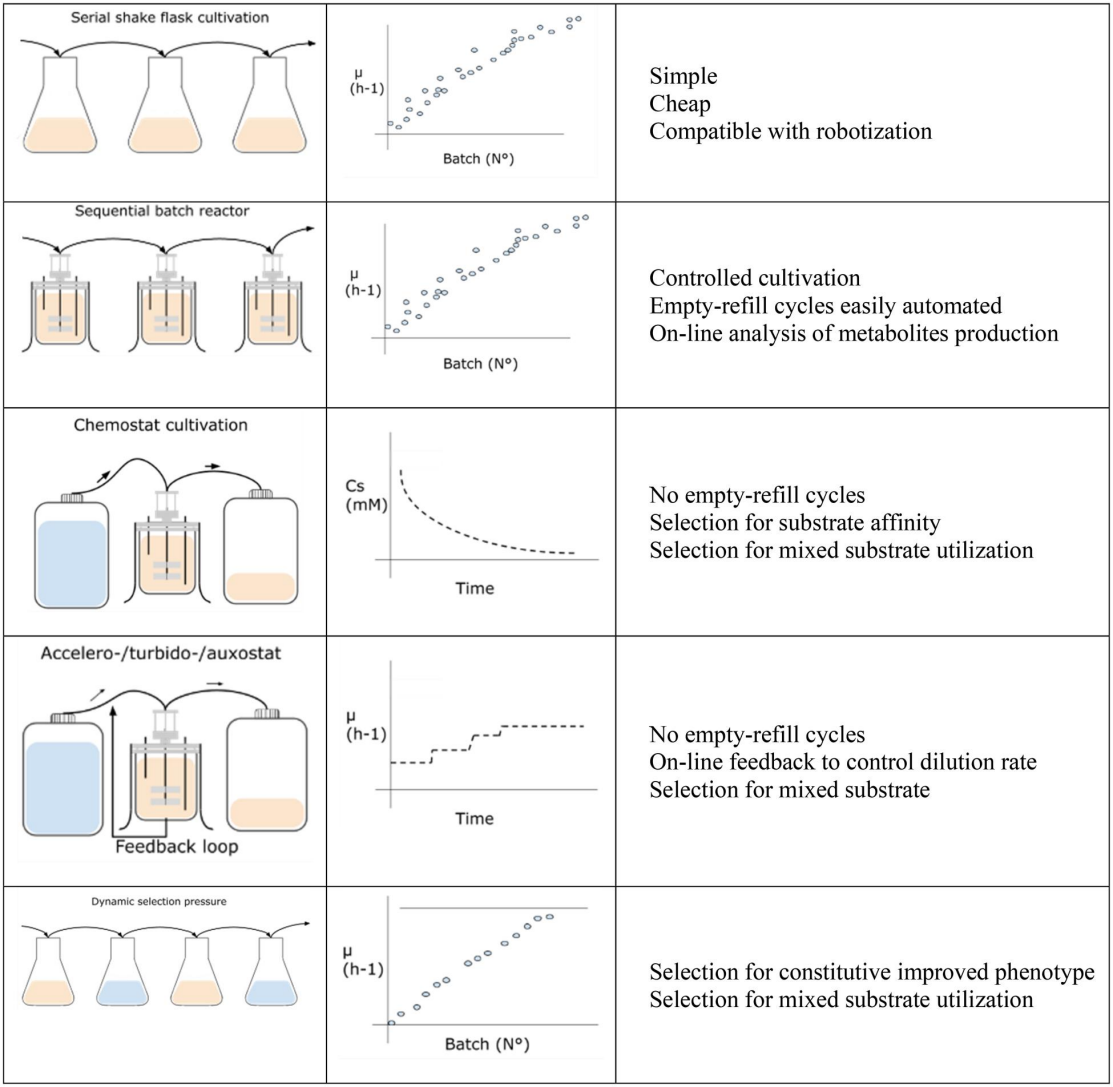
Basic strategies for microorganism cultivation and selection (adapted from [36]). (This figure and all the following have been created using Microsoft Paint and Google Drawings diagramming software).

Screening for improved mutants is a time-consuming task if rudimentary methods are used: for example, growing microorganisms in flasks or microwell plates (Fig. 1) [36]. While methods work well for testing and comparing strains with each other, higher throughput is always desirable. For increased throughput, one of the more promising techniques is the cultivation of mutants in microdroplets. Each microdroplet becomes a separate microreactor for a cell. The benefits of using this technique are increased mutant screening throughput and homogeneous genotype per tested sample. Low growth, but high-yield mutant identification and selection is possible [37].

The first method of Fig. 1, serial transfer cultivation, is simple and cost-effective and has produced yeast strains that have increased stress tolerance, such as high temperature and pH, improved product yields, and increased substrate consumption. The method applies constant or escalating environmental pressure and selects mutants for higher specific growth rates. While the method is cost-effective, it requires manual labor, which takes time or robotization, which is a high-cost investment. Another strategy is the use of batch reactors instead of vessels, such as test tubes and flasks, which add benefits of automation and data from sensors such as CO_2_, pH, oxygen levels, etc. Chemostat cultivation instead of selection for mutants with high specific growth rate uses selection for high substrate affinity, as biomass with metabolites are continuously removed. Similarly, auxostats and turbidostats do not require empty-filled cycles, but instead of selecting on higher substrate affinity, again, selection is based on a specific growth rate as biomass is added back into a bioreactor. Lastly, the dynamic selection pressure cultivation method can obtain mutants with many phenotypical changes simultaneously as each selective pressure is applied in a cyclic approach.

**Figure 2.**
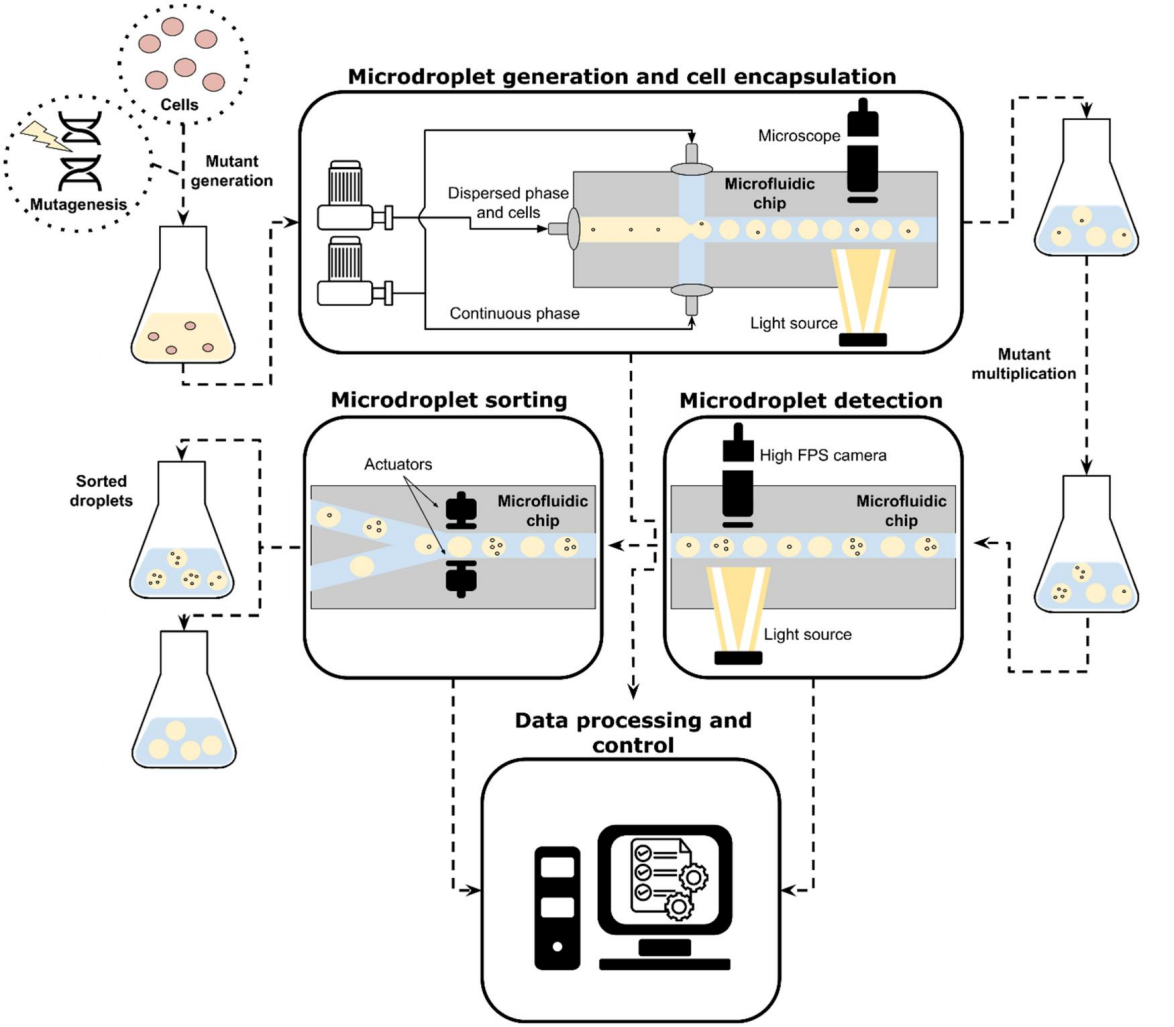
Generalized scheme of the selection of yeast mutants.

The scheme of the entire process of mutant selection using microdroplets is presented in Fig. 2. Water-based media with cell suspension after mutagenesis is encapsulated into droplets, forming an emulsion. Later, droplets with cells inside are incubated (mutant multiplication). Mutagenesis and, for example, possible directed evolution and incubation processes for different microorganisms in this article will not be further discussed. However, we will touch on the stability of emulsions and peculiarities of microdroplet generation as both are connected. It is worth noting that incubation could be performed in a microfluidic chip (MFC) or outside it. Microdroplets must be reinjected into the chip after incubation for selecting droplets with desired cell properties. This requires the detection of droplets and sorting them from the array. Most probably, both of those processes will occur on one device shortly after the other. And microscopical observation for controlling each step of the selection process will be necessary.

Some important things that must be considered in the case of microdroplet technology should be noted. The emulsion must be preferably monodispersed instead of polydisperse. Monodisperse droplets of the same size provide the same growing conditions for encapsulated cells, while polydisperse do not. The required droplet size depends on microorganisms’ size and required incubation time [37]. As soon as cells consume substances from water-based feeding media, the droplet size must be large enough that the changes in concentration will not affect growth. Compatibility of feeding media with base liquid is also important: some compounds from the inner volume of the droplet may diffuse into outer media. Base media may affect the cells’ viability too. Fulfilling these sets of requirements can be challenging, especially in the case of *Y. lipolytica*, as this strain can excrete extracellular lipase and emulsifying agents, which might react with the outer phase [38]. The next microorganism-related aspect is incubation time, which can be from 8 h for fast-growing microorganisms like bacteria [39] to 7 days for slow-growing microorganisms, like algae [40]. With the increase of incubation time and temperature, emulsion stability decreases. Long incubation time can cause oxygenation problems as *Y. lipolytica* and *P. rhodozyma* are aerobic microorganisms. Consequently, one of the best solutions is the use of media with high oxygen permeability [41] or use constant mixing and oxygen supply [42]. However, mixing is not widely utilized because it can create strong shear forces and may destabilize the emulsion. Droplet volume can change during incubation due to microorganism metabolism and some other factors, such as diffusion [43]. This may also affect the emulsion stability during incubation. The reduction of volume can increase the risk of stress associated with high cell-density cultures, such as oxidative stress and changes in glutathione metabolism [44].

Both microdroplet generation rate and cell encapsulation precision have to be as high as possible. On the one hand, we must increase the total number of cells due to low mutation efficiency. However, large numbers or high concentrations of cells may cause difficulties. The first relates to encapsulation: distribution of cells inside droplets may vary from 0 to several cells per droplet. First, droplets must be correctly distributed: one cell per droplet is the optimal condition for further interpretation of the experiment. The other one relates to the generation, detection, and separation rates. The process rates at each experiment step affect the final selection time. Simple calculations show that reaching the desired number of sorted droplets can take tens of hours. Process rates are limited by pressure, such as flow velocities and the speed of detection/sorting devices. Nowadays, a significant increase in throughput from the first published microdroplet generation with 10^2^ droplets per experiment to the current average throughput of 10^2^–10^3^ droplets/s and even reaching 10^4^ droplets/s generation has been achieved.

Finally, both the detection and sorting methods of microdroplets must be sensitive and fast enough for mutant selection. Currently, those techniques utilize electric fields, acoustic waves, magnetic fields, laser heating, etc. [45].

Though microfluidic technology has been advancing at a high pace, there are still prominent hindrances. Chip design and manufacturing are still expensive and not easily available in comparison to 96-well plates. Expenses add up, considering that micropumps, flow-microcontrollers, and specific miniaturized devices for the detection and separation of microdroplets are also required. Finally, the optimization process can be difficult and require a lot of refinement before studies begin [46]. Nevertheless, technology is promising, and one may try to find ways to overcome drawbacks.

## Designing of microfluidic system

Deciding if the microdroplet system will work for the project requirements is challenging as the microdroplet system has many interlinked factors and changing one factor will impact the entire system. Here we will discuss correlations between several factors to better understand and design the entire microfluidic system.

### Microdroplet generation and cell encapsulation

**Figure 3.**
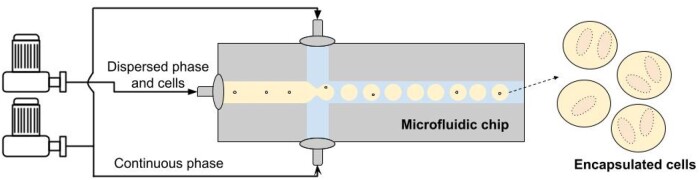
General idea of emulsion formation.

As it was mentioned earlier, the idea of emulsion formation is well-known and quite simple—mixing two liquid phases that are insoluble in each other, one of which is continuous, and the other serves as a dispersion medium containing cells (see Fig. 3). The process of droplet generation could be characterized by desired droplet sizes, their variability, and a generation rate.

Fluidic flow is established with the help of one or several micropumps or pressure controllers and with a device for controlling fluids as well as for the detection and separation of droplets, shortly called a MFC. As soon as the flow is not constant, variability of droplets’ sizes appears. Nowadays, pressure-controlled pumps are most frequently used as they have the smallest fluid pulsation in comparison to syringes and peristaltic pumps [47]. This allows us to obtain droplets with the smallest differences between them, generating monodisperse emulsion to provide unified growing conditions. Those differences could be characterized by the coefficient of variability (**CoV**) of droplets’ sizes (Equation (1)):

(1) $$\mathrm{CoV}=\frac{\sigma }{\mu },$$

where ***σ*** is the standard deviation of droplet sizes, and μ is the average size of droplets.

The generation rate depends on droplet size and is affected by total flow velocity and properties of liquids. On the one hand, the flow velocity is limited by the total hydrodynamic resistance of the microfluidic systems, including supplying tubing, which has a pressure limit that both the controller and chip can hold. On the other hand, increasing flow velocities will change the flow from laminar to turbulent until the moment when droplet generation fails. Finally, droplet dimension depends on the flow ratio between used liquids, their properties such as viscosity, materials’ surface characteristics, and channel geometry of MFC [48]. The relationships between the above-mentioned parameters are also shown in Yao *et al*. [49]. Chip design has an extremely wide variety of options and the final configuration depends on many factors. In this section, some of them and their relation to the full process will be discussed.

#### Geometry and design of microchips for droplet generation

As shown, for example, in [48], the size of microdroplets depends on the microchannel dimensions and flow rates. The size of the droplets increases with the increase in the size of the microchannel but decreases with the increase in the flow rate of the continuous phase.

**Figure 4.**
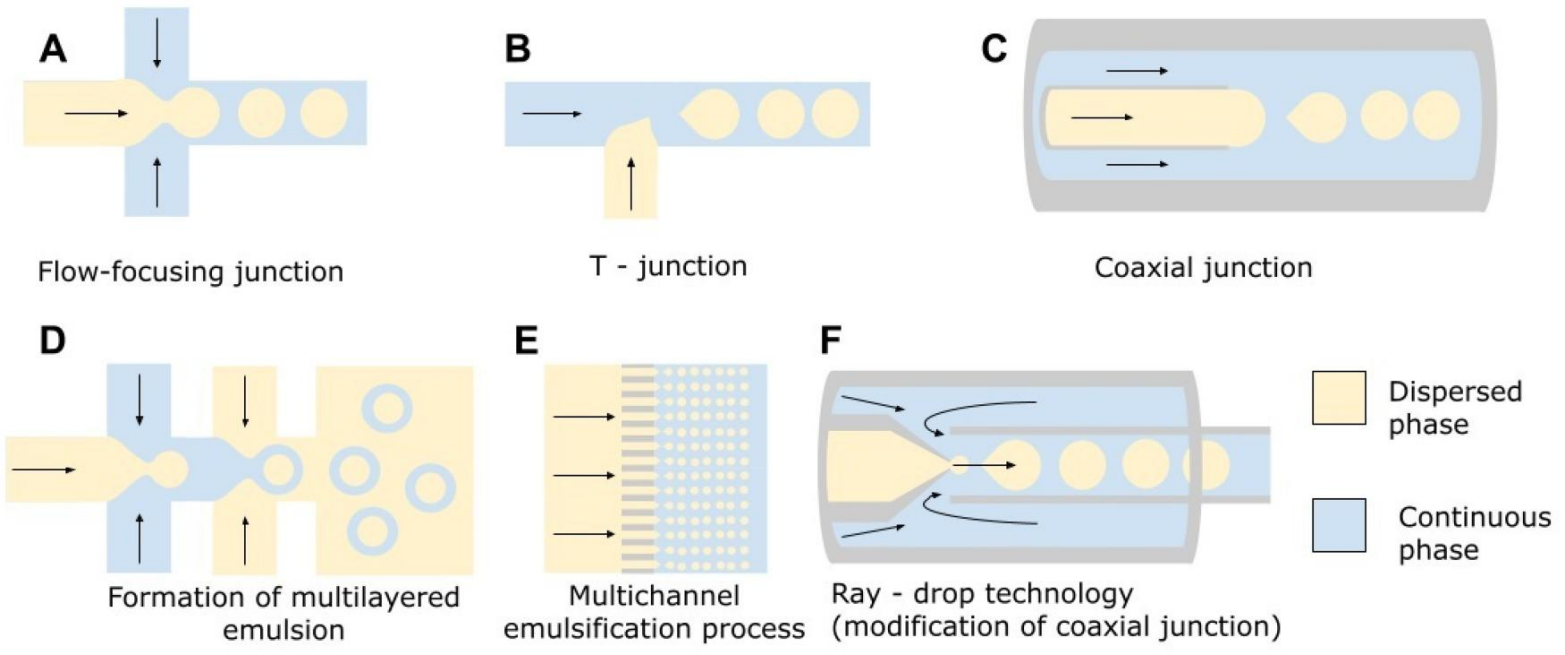
Typical microfluidic geometries for creating microdroplets: (A) flow-focusing; (B) T-junction; (C) coaxial junction; (D) formation of multilayered emulsion; (E) multichannel emulsification process (images adapted from [51]); (F) ray-drop technology (image adapted from [57]).

Nowadays, the methods of controlling the process of droplet generation or fluids’ flows at the moment of formation of droplets could be classified as passive and active [50]. A review of passive approaches focuses on the characteristics and mechanisms of droplet formation modes occurring in microfluidic channels of specific geometry [50]. Some typical microdroplet passive generation designs [51] are shown in Fig. 4: flow-focusing, T-junction, co-flow, and multichannel emulsification configurations, etc. Active approaches cover state-of-the-art techniques employing external fields. Acoustic [52, 53], electrical [54], mechanical [55], and thermal [56] forces can be locally applied to tune droplet dimensions and production rates.

Methods like flow-focusing or T-junction are simpler for controlling droplet sizes. The multichannel method can have the highest productivity when considerable number of droplets is needed, but also has difficulties with adjusting flows—the smallest differences between channel walls or flow instabilities can cause greater in comparison to the single-channel method variability of droplet sizes. Also, it requires controllers with high-pressure limits.

**Figure 5.**
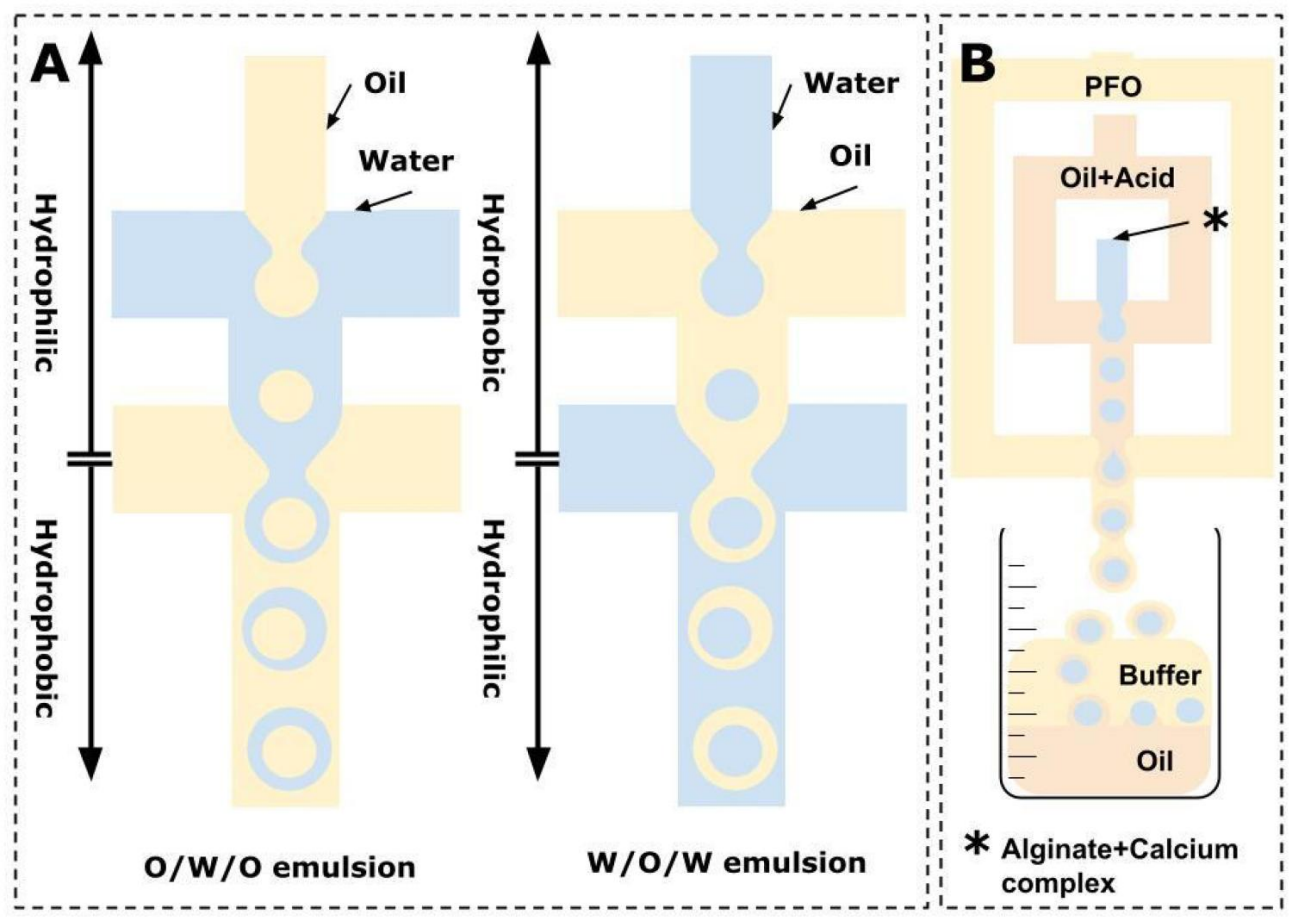
Formation of complex emulsions (A) (adapted from [58]) and capsulation of droplets (B) (image adapted from [59]): (A) scheme reveals the importance of hydrophobicity (hydrophilicity) of channels during the stable formation of o/w/o or w/o/w emulsions (see also [60]); (B) alginate with calcium complexes is injected in flow-focusing junction that contains oil and acid, which starts gelation reaction. Formed droplets are injected in the second flow-focusing junction containing PFO, which destabilizes the emulsion. Microgels are then transferred from the oil to the water phase.

Those basic structures can be supplemented by additional channels to create more complex emulsion (see Fig. 5A) [58] or encapsulation of cells using alginate gelation (Fig. 5B) [59].

#### Chip material

Nowadays, there are many materials available for the creation of microfluidic devices, starting with silicon-based chips [61], chips based on glass, ceramic materials and metals [62–65], polymers [66–69], hydrogels [70–72], and composite materials [73–75]. The choice of the chip material can be determined by the desired type of emulsion [51, 76]. The hydrophilicity or hydrophobicity of the chip channels should be considered for a stable generation process: for water-in-oil (w/o) droplets, the channels must be hydrophobic (see Fig. 5A). For example, in the case of glass that is a hydrophilic chemical modification must be applied to make channels walls hydrophobic. The subsequent methods of detecting and separating microdroplets must also be considered: optical transparency, absorption at some specific wavelength range, or the need to make channels in the submicron scale. One can refer to references [77] and [78] where some properties and typical applications of material in microfluidics are mentioned. The technological aspects of chip manufacturing go far beyond the scope of this article. However, here we mention some reviews that are devoted to the latest progress and perspectives in the field [78–81].

Polydimethylsiloxane (PDMS) and glass are widely used materials that are the most biologically compatible, inert, and suitable for repeated use. It is worth noting some peculiarities when using those materials. It was found and reported that PDMS can leach, and hydrophobic compounds (proteins, or small molecules, like fluorescent dye Nile red) could be absorbed on chip surface [82–84]. Also, the structure of this material makes it gas-permeable (which is an advantage) and makes the evaporation of water easier at the same time. Those effects have a strong influence on the chip cell cultivation process. And, also, leaching and sorption can make additional noise during detection. Some techniques were investigated to overcome these issues, like modification of chip surface or addition of specific oligomers before polymerization [85–87]. However, some of these steps are not easy to perform, and the desired effect is temporary, as reported. A flexible PDMS chip can change shape under the pressure of the liquid, which might be crucial for the chosen specific geometry. Also, all mentioned changes in material properties can make microfluidic processes unstable in time. Glass does not have some of the above-mentioned drawbacks and can hold higher pressures in comparison to PDMS. However, such kind of chips are much more expensive due to more difficult manufacturing process, they are fragile and have zero gas permeability.

The choice of material for MFC should be done considering droplet detection and separation methods, for example, at the stage of separation of single-cell droplets before incubation, which will be discussed in the next sections.

#### Properties of liquids, emulsion stability, and cultivation

First, the emulsion must be optimized in terms of cell cultivation conditions. Depending on which phase is dispersed and which is continuous, several types of emulsions could be produced and used in microfluidics (dispersed/continuous phases): w/o, oil-in-water (o/w), and multilayered ones (w/o/w, o/w/o, etc.). Feeding medias for *Y. lipolytica* and *P. rhodozyma* are water based. That is why the desired types of emulsion could be w/o or w/o/w.

On the one hand, we must find the best feeding media and conditions for cell growth, which does not guarantee the formed emulsion will be stable. On the other hand, the stability of the generation process and the formed emulsion does not mean optimal conditions for cell growth and their viability.

W/o emulsions are usually much less stable than o/w due to the high interfacial area of the dispersed phase. Overtime emulsions can self-separate into two phases through different mechanisms: creaming, coalescence, flocculation, or Ostwald ripening [88]. Usually, the stability of the emulsion is determined visually, microscopically, by particle size analysis, or by charge analysis and rheology measurements [88]. Views on the nature of w/o emulsions were published in [89–93]. Stability factors include oil viscosity, temperature, droplet size, pH of the aqueous medium, presence of modifiers, etc. Besides, some factors, such as changes in the concentration of substances during the incubation process due to cell activity and semipermeable droplet walls, should be considered. Some viable solutions that cover the mentioned difficulties include the addition of specific biocompatible stabilizers [94–97], the formation of alginate capsules [98–101] (see Fig. 5B), or complex multi-layered (w/o/w, etc.) emulsions [58, 102–104] (see Fig. 5A).

A lot of researchers in the field of microfluidics use fluorinated oils as a continuous phase because of their inertness, biocompatibility, and gas permeability. The latest progress on the stability of fluorinated oil emulsions and some specific surfactants was reported in [105–108]. The surface-active biocompatible additions and stabilizers w/o emulsions that are commonly used for emulsion stabilization are polysaccharides, proteins, and chemically synthesized compounds, like Span 80, Tween 80, or polyglycerol polyricinoleate, etc. [90]. Surfactants could be added both to o/w phases as well. Their concentrations may vary in wide range (up to 16% mas. [97]) and allow to get emulsions with droplet sizes up to 100 µm [109]. Emulsion could be stable even for 15 days at 50°C (for 40 μm droplets) in some cases [110]. With such time stability, microdroplets seem to be suitable for the cultivation of *Y. lipolytica* [111]. The majority of available data indicate two fluorinated oil options: Novec™ 7500 [39, 112–115] or Fluorinert™ FC-40 [40, 116]—it has been tested and demonstrated that microdroplets can be successfully stored on a chip even for 10 days [116].

In some cases, capsulation of droplets for the cultivation of microorganisms could be applicable [117, 118]. The process of capsulation of droplets has drawbacks, such as non-spherical droplets, higher costs of production, toxicity of some organic solvents, and droplet agglomeration [119].

The last-mentioned multilayered emulsions could be used for the selection of slow-growing microorganisms in low-nutrient environments [120]. However, using complex multilayered emulsions is difficult due to challenges in their formation, stability, and reproducibility [93].

In each case, the search for the optimal conditions for cell growth and stability of emulsion and features of droplets’ formation seems to be an independent experimental task. Also, it is difficult to determine the desired droplet size for incubation from the first principles. However, one may look at research on the successful cultivation of other similar microorganisms. One of the most widely researched organisms is *Escherichia coli*, and microfluidic technology is no exception. Droplet size used for *E. coli* experiments varies between 10 and 20 µm [39, 121, 122]. Another often-used microorganism for microfluid technology is algae, with microdroplet sizes used ranging from 50 µm for *P. tricornutum* [40] to 80 µm for *Chlorella vulgaris* [116]. While yeast microdroplet sizes vary from 30 µm for *Y. lipolytica* [115] to 65 µm for *Saccharomyces cerevisiae* [114]. We may suggest the droplet size suitable for yeast cultivation might be in the range of ∼30 µm at the beginning of experiments. These initial conditions for droplet generation should also include oxygen-permeable fluorinated oil with surface-active modifiers such as Pico-Surf™ 1 [114], EA surfactant [112], or FluoroSurfactant 008 [123].

In addition, the need for later safe cell release emulsion breakdown after the microdroplet separation process should be considered as well [124].

#### Cells encapsulation

The number of droplets in the emulsion depends on the rate and time of the generation process. Both generation frequency and time are limited. On the one hand, generation frequency is limited by physical factors that are discussed above. On the other hand, time for generation has to be limited to prevent cell division before incubation, or some steps should be taken to stop the proliferation. Moreover, the number of mutants to be screened and, consequently, the number of droplets depend on mutagenesis type and cultures’ pre-treatment.

The frequency of mutations significantly depends on the mutation factor and microorganism and varies a lot. For example, it can reach 4.55 × 10^−14^ per gene per cell for *UV-*mutagenesis [125] and 1.17 × 10^−6^ per gene per cell for EMS [126]. The probability of the desired mutation remains unknown. Differences in mutation rates between wild-type yeast species vary from 1.1 × 10^−7^ to 5.8 × 10^−7^ [127]. Some simple calculations show that the initial cell number, considering the survival rate, is approximately 10% after EMS mutagenesis and the number of genes of *Y. lipolytica* is ∼ 6 × 10^3^ [128], which must be ∼10^4^ to give at least one mutant for encapsulation. From this point of view, increasing the number of droplets with mutants for further investigation to ∼10^6^–10^7^ requires ∼10^10^–10^11^ cells at the beginning of the experiment. However, the concentration of cells for encapsulation affects the process itself.

Each droplet should contain only one cell so that the characteristics of each microdroplet after incubation may be attributed to the behaviour of a single cell rather than an array. It has been shown that when cells are randomly distributed in a carrier solution, cell encapsulation follows a Poisson distribution [see Equation (2)]:

(2) $$P\left(X\right)=\frac{\lambda^{X}e^{-\lambda }}{X!},$$

where ***P*** shows the fraction of droplets that will contain ***X*** cells. ***λ*** is the mean number of cells per droplet and is calculated by multiplying cell concentration by the droplet volume.

The encapsulation efficiency of cells inside droplets of a given size can be calculated using an online calculator [129], based on the initial concentration of cells. Occupation efficiency is around ∼12% for 30 µm single-cell droplets, and ∼87% of them will be empty at the concentration of 10^7^ cells/ml. Some droplets (∼1%) will contain two or more cells inside as well. The latter effect could be even higher, considering that cells may adhere to and aggregate on channel walls [130]. It becomes more concerning as cell culture concentration increases.

Working parameters of the above-mentioned Ray-drop technology (Fig. 4F) are as follows: for w/o droplets with a size of ∼71 μm at a generation rate of ∼445 Hz, flow rates equal to 100 μl/min and 5 μl/min for continuous and dispersed phases, respectively [131] (operating pressure was not specified). So, considering the efficiency of encapsulation, 8 × 10^6^ droplets with approximately 10^6^ mutants inside could be generated after 5 h. Initial cell concentration varies a lot: from 3 × 10^6^ cells/ml [39] to 4 × 10^8^ cells/ml [121], as reported.

The process of encapsulation could be optimized. It might be useful to add some stabilizers to prevent cell aggregation in dispersed phases, like OptiPrep [114], or use methods of manipulation of cells to increase single-cell encapsulation efficiency or to sort out the single-cell droplets. Different methods and techniques were developed for those purposes. An overview of the so-called ‘active’ and ‘passive’ methods for the optimization of the encapsulation process can be found in references [77, 132–136]. As an example, authors [137] improved the cell encapsulation efficiency with a cell size of 14 µm by 72% using the flow-alignment technique.

Since, at this stage of experiment planning, a lot of initial parameters became known, such as the desired size of microdroplets, liquids and chips’ material properties, the method of generation, dimensions of the channels, flow rate of liquids, and frequency of generation can be estimated and adjusted from the initial data. Currently, there is software that allows modelling the final geometry of a microchip from the first principles [138]. This is also needed for the estimation of the total hydrodynamic resistance of a microfluidic system containing both droplet detection and separation parts. Several online tools [139–142] are available to estimate flow parameters for specific channel geometries. These tools also help to calculate some important MFC and hardware characteristics, such as controller and flow sensor operating parameters. One can refer to original process modelling papers [143–147] as well.

### Microdroplet detection

The next step for the successful selection of microorganisms is to distinguish microdroplets with the desired cell properties from one another. In other words, one must find a suitable marker. Metabolization of cells and proliferation may change droplet size and physical–chemical parameters of feeding media: changes in concentration of initial components and the appearance of internal or external metabolites (astaxanthin, in the case of *P. rhodozyma*; lipase, in the case of *Y. lipolytica*). Consequently, those affect changes in the optical density and electrical conductivity of droplets. Also, the possible appearance of several dozen cells inside a microdroplet after incubation could be interpreted as a mass/concentration heterogeneity—droplets of different sizes and number of cells inside will have different densities. In our case, the choice of detection method will be limited to the use of non-destructive methods without any labelling of cells, as it is undesirable for further use of mutants.

The reviews [148–153] summarize progress in detection methods used in microfluidic droplet systems and discuss the advantages and disadvantages of their use in different cases (some of them are presented in Table 1 [154, 155]). These methods include optical, electrochemical, mass spectrometry, etc. Some less commonly used techniques are published in Ref. [156]. Here we will discuss some methods that seem to be the most promising and suitable for our yeast selection experiments, considering the above-mentioned markers.

**Table 1.** Analytical detection techniques for droplet microfluidics [154, 155].

Analytical method	Sensitivity	Analysis speed	Labelling	Advantages	Disadvantages	Applications
Bright-field microscopy	Poor	Depends on camera speed	No	Imaging the shape, size, colour, trajectory of droplets, and interaction between droplets	Low sensitivity, poor ability for molecule analysis	Droplet generation and manipulation

Fluorescence microscopy	Good	Fair	Yes	Convenient; Good sensitivity; Quantification	Low analysis speed; Need derivatization	Single-cell and single-molecule analysis

LIF	Excellent	Excellent	Yes	High sensitivity; compounds quantification; Large dynamic range	High dependence on fluorescent probe design	Single-molecule detection; single-cell analysis; high-throughput sorting and screening

Laser Raman spectroscopy (LRF)	Good	Good	No	Good sensitivity	Matrix effect	High-throughput detection; bacteria identification

Absorption spectroscopy	Poor	Good	No	Label-free; compounds quantification	Low sensitivity	Rapid identification of compounds

Mass spectrometry	Good	Good	No	Simultaneous detection of multiple analytes; Capability of elucidating chemical structures	Matrix effect; Requiring sample pre-treatment	Screening and optimising reaction conditions; enzyme inhibitor

Photothermal- and optoacoustic spectroscopy	Good	Good	No	Compounds quantification	Matrix effect	Biomedical diagnostic tools; enzymatic assay

FTIR spectroscopy	Good	Good	No	Simultaneous detection and quantification of multiple analytes	Requiring sample pre-treatment; matrix effect	Chemical reactions study

Nuclear magnetic resonance spectroscopy	Poor	Poor	No	Capability of elucidating chemical structures	Requiring sample pre-treatment	Rapid identification of compounds

Electrochemical detection	Good	Good	Yes	Low cost; Simple structure; Small size; Good sensitivity	Need derivatization, poor reproducibility	Measuring the size, frequency, and velocity of droplets

Impedance-based detection	Good	Good	No	Low cost; Simple structure; Small size	Matrix effect	Measuring size, number of cells inside droplets

Capillary electrophoresis	–	Fair	Yes	Separation of multiple analytes	Need derivatization with fluorescence detection	Separation of amino acid; enzyme assay

#### Droplets’ size

**Figure 6.**
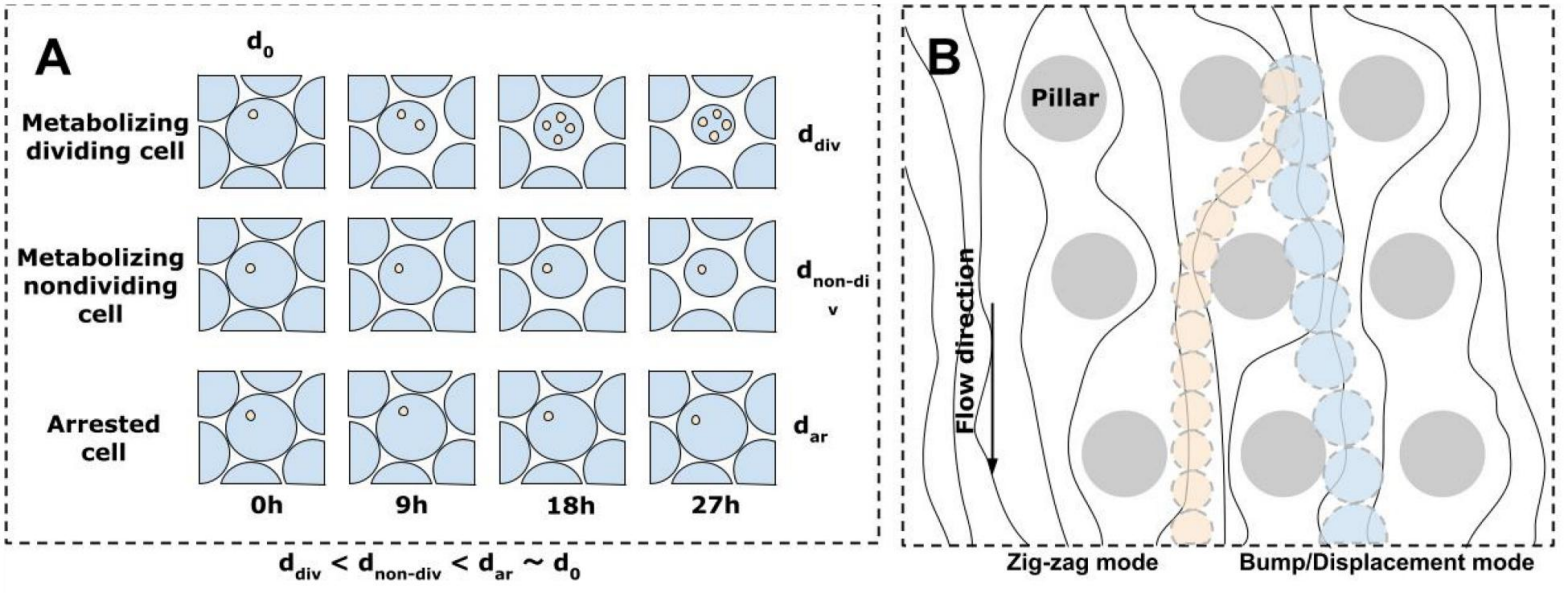
Cells behaviour during incubation (A) (image adapted from [43]) and principal scheme of DLD sorting method (B) (image adapted from [171, 172]): (A) Droplets’ size reduction due to different cell metabolism and division: at the beginning, all droplets are uniform in size; the largest change is caused by metabolizing and dividing cells, smaller changes are caused by metabolizing and non-dividing cells, while arrested cells do not cause droplet size reduction; (B) two separate modes of particle flow types through chip—zig-zag and bump/displacement mode. The chip contains pillars distributed with a horizontal shift to produce a periodic streamline pattern in laminar flow. It causes smaller particles to flow through pillars, while larger particles are more likely to bump, which displaces them in one direction.

Obviously, if droplet size changes after incubation, it could be noticed and measured microscopically. As shown [157], the difference in volume between ‘empty’ (without cells) and inoculated hydrogel droplets was significant after cultivation. The equivalent at the beginning of incubation, empty droplets remained the same at ∼144 pL and inoculated by *S. cerevisiae* droplets size was ∼65 pL, which is equivalent to 79% of the initial diameter. Growth-induced droplet size-decreasing phenomena were observed for various microorganisms as well [158–161]. As mentioned by the authors [160], this phenomenon depends on several factors, like droplet volume, medium content and concentration of substances, number of cells, incubation time, and osmosis (see also Fig. 6A).

On the other hand, there are inertial-based methods for particle separation already known in industry, and the effects of such techniques were revealed in microfluidics, too. This part of microfluidic technology is known as inertial microfluidics—a description of it and the occurred effects can be found in [162–169]. Methods developed on this basis are known as passive methods. And particles with different characteristics under inertial forces are self-detected and separated at the same time. Here we discuss some methods that partially touch separation techniques too.

**Figure 7.**
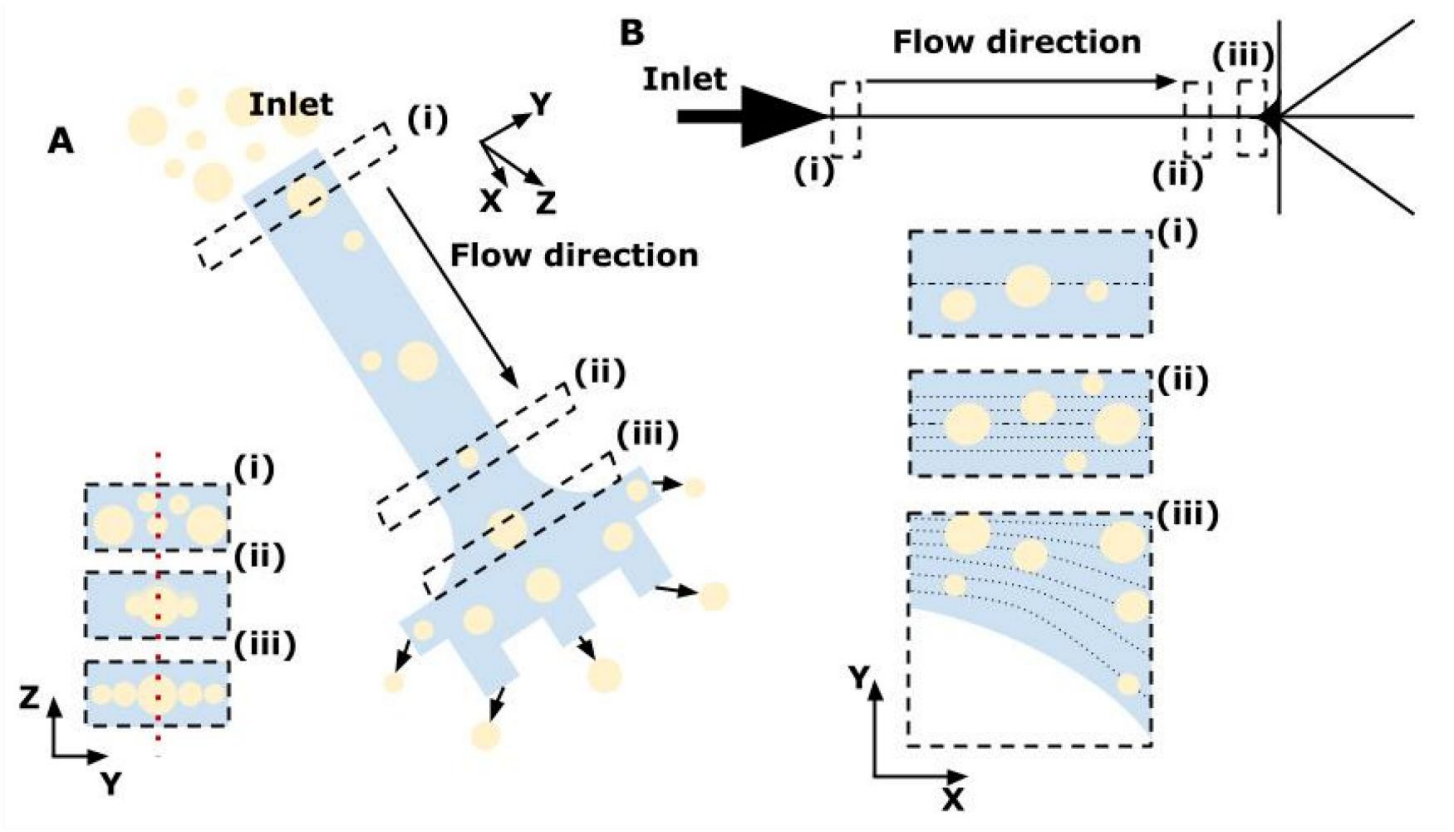
Size-based separation of droplets due to lateral inertial focusing (image adapted from [158]). (A) A view of an inertial microfluidic channel. (B) Scheme (upper) of the microfluidic device with outlets O1–O5 and illustration of the lateral distribution of droplets of different sizes (down) at three indicated regions (i), (ii), and (iii).

A technique known as deterministic lateral displacement (DLD) [170, 171] (see Fig. 6B) was used by the authors [172] for the separation of ∼23 µm droplets with *S. cerevisiae* inside from ∼30 µm empty droplets at a rate of approximately 12 000 droplets per second. Other authors [158, 173] used viscoelastic effects for sorting, too: after the incubation period, empty droplets were successfully separated from encapsulated *E. gracilis* droplets with efficiency of 93.6% (see also Fig. 7) [158].

Currently, DLD devices are developed through numerical simulation and experimental validation [174, 175]. However, some crucial factors must be known. One may suggest that droplets with different proliferation activity of cells may have varied sizes after cultivation (multimodal droplet sizes). Also, small experimental data were published to predict how big those differences could be after cultivation in the cases of *Y. lipolytica* and *P. rhodozyma*. This means the DLD chip must be adjusted and developed twice. Moreover, cells may consume the medium without division, also resulting in a decrease in droplet sizes [176] (see Fig. 6A). Some experiments on cell behaviour during incubation inside droplets must be performed to predict the possibility of this method’s implementation. Nevertheless, the mentioned drawbacks could possibly be covered by methods that allow multimodal particle sorting [177–179].

**Figure 8.**
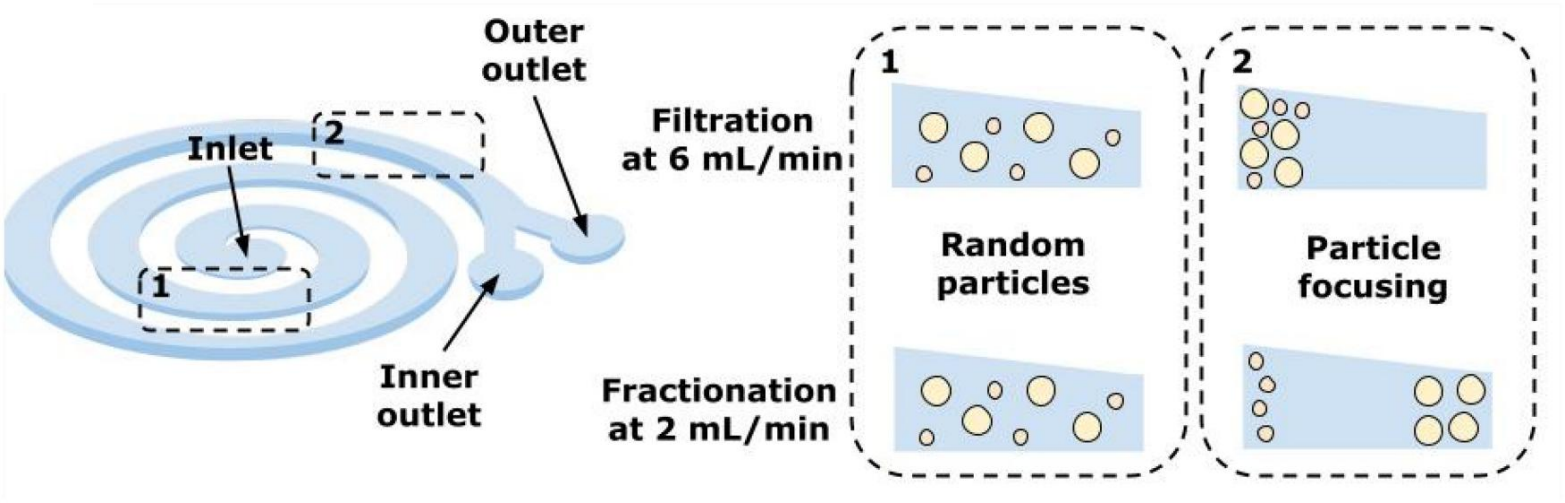
Inertial focusing of particles (image adapted from [166])—with a random order of particles at the beginning (to the left, 1), one may reach filtration (to the right, 2 top) or fractionation (to the right, 2 down) by adjusting flow velocity. Separation is achieved through Dean drag and inertial lift forces in the channel.

The inertial forces that arise during the movement of liquid in channels of a special geometry contribute to the movement of particles immersed in it in the cross-section of the channel [166, 180] (see Fig. 8). Thus, the particles/droplets are concentrated near one of the walls of the channel, where they can be detected and collected in some way later.

#### Optical and spectroscopic detection methods

Remain the most widely used due to their versatility, contact-free, and non-destructive nature, but may have a low signal-to-noise ratio [152, 181–183]. The classical adsorption method of biomass concentration measurement as optical density has long been known and widely used. However, adsorption and fluorescence, when moving to the microscale, significantly lose sensitivity due to a significant reduction in the length of the optical path. There is an example of work where the optical path was increased by partial deformation of the droplet and a change in the geometry of the channel (Z-channel) [184]. Also, there are techniques known as flow cytometry (FC) [185–187], fluorescent-activated droplet sorting (FADS) [188–190], or laser-induced fluorescence (LIF) [191] for single-cell/droplet analysis and sorting. Those techniques utilize lasers on one or several wavelengths to produce scattered and fluorescent light signals from a single unit. If one can make droplet fluorescent, this increases sensitivity of detection and selection.

As mentioned, internal metabolite—astaxanthin has strong fluorescence, and this property was used for the successful selection of carotenoids hyperproducing *P. rhodozyma* [192] and *Xanthophyllomyces dendrorhous* [193] mutants. The laser tweezers Raman spectroscopy was used for rapid quantification of total carotenoids in single cells of *Rhodotorula glutinis* [194] as well. This method is sensitive to the chemical composition of the cell [195].

Increased production of biomass of astaxanthin-producing strain of *P. rhodozyma* is of interest, too. When using only astaxanthin production as a biomarker, one should consider that droplets with high biomass-producing strain may have the same (or lower) fluorescence intensity as astaxanthin-hyperproducing strains with lower biomass content. Nevertheless, fluorescence could be used as an additional factor for further selection of sorted droplets: high biomass production strain with exceptionally low astaxanthin production is out of interest.

Droplets can be made fluorescent when an extracellular compound reacts with a specific reagent and forms a fluorescent product. For example, high ethanol-producing cyanobacteria was successfully selected in microdroplets by pico-injection of an enzymatic assay. It converts ethanol into a highly fluorescent compound, resorufin [196]. In the case of *Y. lipolytica*, the concentration of extracellular lipase might be measured using the same technique by lipolysis of the *p*-nitrophenyl esters that give yellow-coloured *p*-nitrophenol, measured at 405–410 nm [197]. For example, strains of *Y. lipolytica* with high-active heterologous enzyme secretion were studied using BODIPY^®^FL: hydrolysis releases fluorophores, revealing enzyme activity inside droplets [115]. Another example [198]: the population of *Bacillus subtilis* was screened by decreasing droplet fluorescence as soon as enzymes were able to hydrolyse a fluorogenic substrate, already present in media.

Also, a method of co-cultivation of microorganisms was used for selection of droplets: one developed a FADS co-culture pipeline for improving erythritol production in *Y. lipolytica*: the pico-injection of fluorescence-based erythritol-biosensing *E. coli* was used to make fluorescence droplets [199].

Another optical method, like light-scattering-based droplet screening, could be used for the detection and identification of droplets as well [200]. For example, authors [201] used scattered laser light from nanolitre droplets with different encapsulated cell proliferation activities and demonstrated reliable detection for 12 different bacterial species at 1.2 kHz. Some drawbacks, like high noise levels of scattered light from the w/o surface, could be overcome using special types of oils to make differences in refractive indexes of liquids smaller [202].

The method of optical recognition in the bright/dark field using machine learning (ML) and artificial intelligence (AI) remains the most universal [203–208]. The detection algorithm can be easily modified for different cells/particles without changing other microfluidic system components. Sorting performance is limited by the speed of the optical camera and the image processing in the absence of other limiting factors, such as flow rate and the inertia of the sorting device. As an example, optical recognition and image processing were used for investigations of biomass growth inside droplets by [157, 209, 210], etc.

#### Electrochemical methods

Electrochemical methods could be used for droplet detection as soon as the concentration of substances inside the droplet changes, and this affects electromagnetic conductivity and permeability of media. Methods include techniques like amperometric detection, capillary electrophoresis, conductivity-based detection, cyclic voltammetry (CV), and electrochemical impedance spectroscopy (EIS) [211–217]. They are characterized by obvious advantages: label-free, have high speed of detection, relative ease of manufacturing.

**Figure 9.**
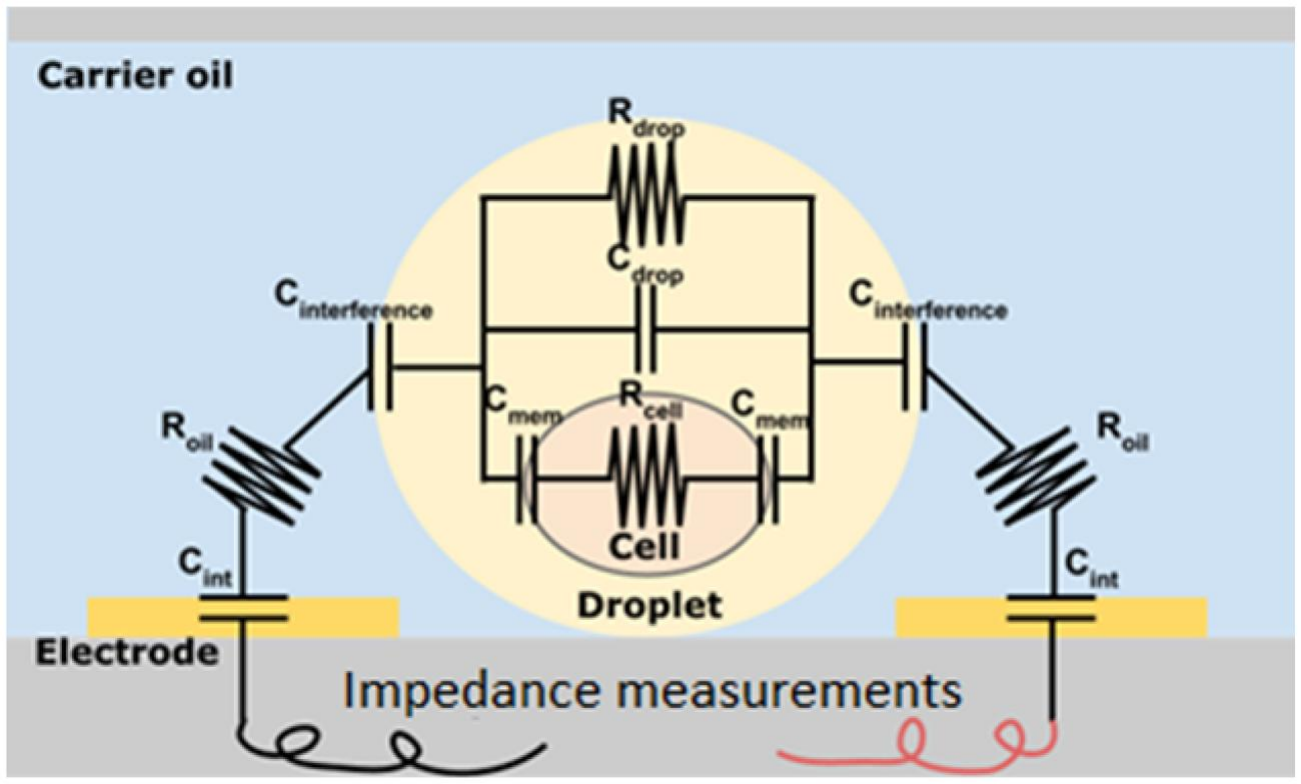
Principal electrical scheme of a droplet in the channel for EIS (image adapted from [220]): C, R—electrical capacitance and resistance, respectively.

As an example, the authors of the paper [213] used differences in the impedance for recognition of droplets with and without cells (see Fig. 9). They were able to distinguish w/o droplets of ∼40 µm even with different numbers of cells inside and showed that sensitivity and reliability of this type of analysis depend on the electrical conductivity of feeding media. If the conductivity of internal media is higher than >1.6 S/m, the difference in impedances becomes negligible. Cultured microdroplets of yeasts are large, and the feeding media can have an electrical conductivity of ∼2 S/m [218], what makes this method difficult to apply when nutritionally rich mediums with various salts are used. However, the ability to improve the encapsulation efficiency makes this method attractive. Cylindrical (wrap-around) electrodes are more sensitive [219], but such a configuration makes the channel non-transparent.

It is worth noting that high-frequency electromagnetic fields should be used with caution as they may negatively affect cell viability [221, 222] due to the instability of cell membranes.

The last-mentioned in this chapter marker is the appearance of *mass/concentration heterogeneities inside microdroplets* in the form of new cells, what affects changes in the droplet centre of inertia and density. And, of course, the appearance of new cells could be detected microscopically as well.

**Figure 10.**
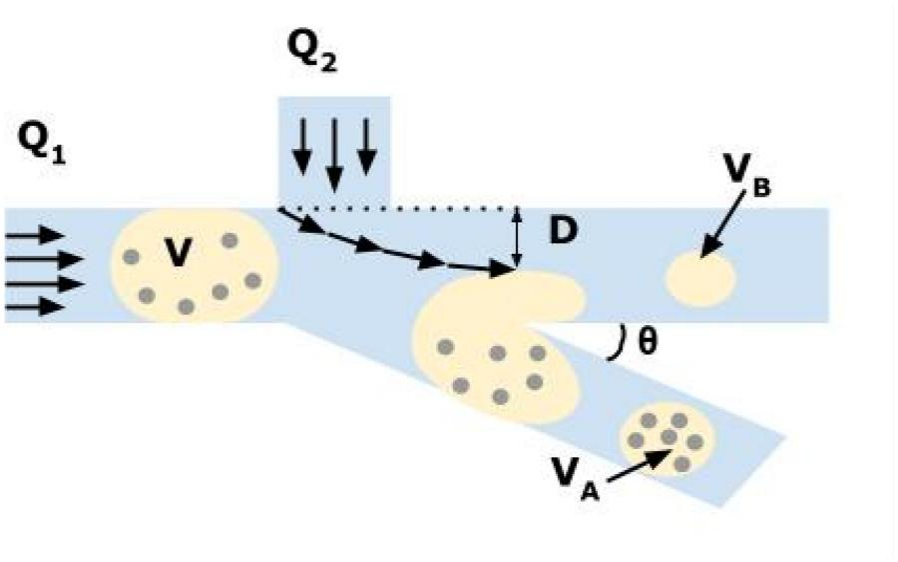
Splitting of the droplet (image adapted from [223])—the sizes of split-off droplets (V_A_ and V_B_) are determined by the flow rate ratio of Q_2_ and Q_1_ in microchannels.

As mentioned above (see Fig. 8), inertial forces can move particles to the channel wall. The same effect touches cells that move to the w/o interface inside the droplet. This gives the possibility to split it into several parts (see Fig. 10) [223]. One can obtain higher cell concentration inside one part of a droplet, and this allows the possibility to analyse the rest of it (the second part) by chromatography, spectroscopy, etc. (see Table 1). This method was used by Tenje et al [224], for example. One may refer to [223, 225–230] where this kind of technique was used too. However, such a technique requires high-precision particle manipulation and storage for referring correlation between two parts of the same droplet.

Finally, it is worth mentioning a density-dependent sorter developed by Nam *et al*. [231]: same size ∼150 μm alginate droplets, but with different number of encapsulated cells inside, were separated using a standing surface acoustic wave at a rate of 2300 droplets per second. This technique is known as *acoustophoresis*. It is suitable for sorting many particles at the same time.

**Figure 11.**
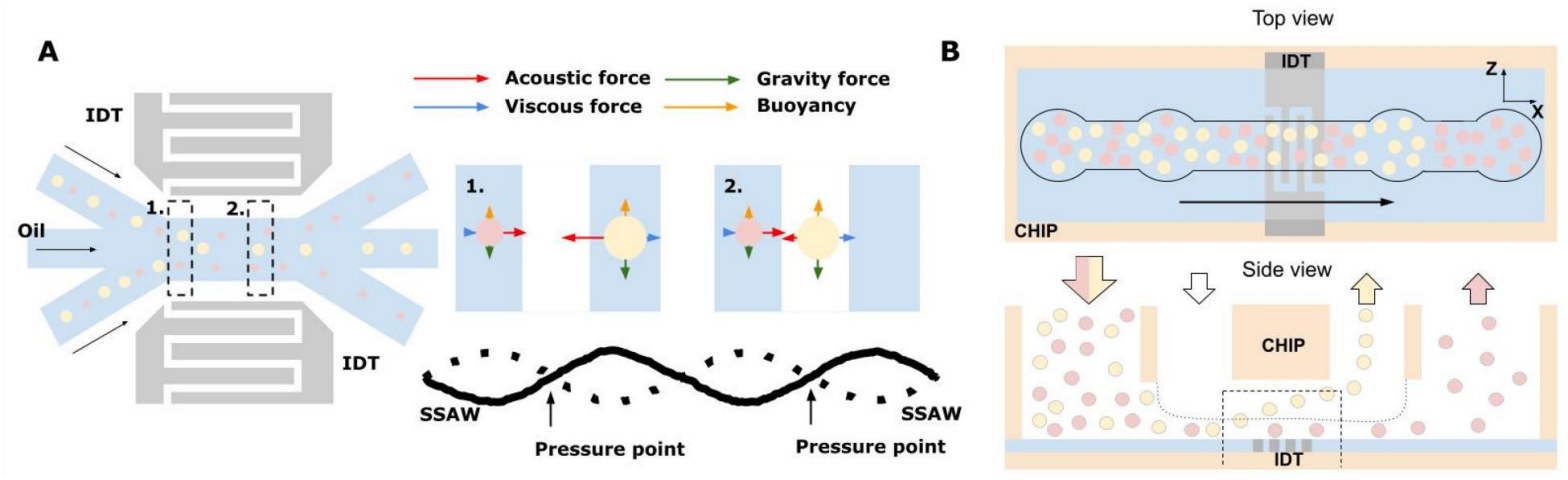
Acoustic methods and their modifications (images adapted from [238, 239]): SSAW device scheme is shown (to the top left), and the separation mechanism is shown (to the top right). (A) Acoustic forces applied from interdigital transducer (IDT) move bigger particles to pressure nodes; (B) an IDT under the microchannel was used to separate polystyrene particles of varied sizes (image adapted from [240]).

Acoustic pressure applied to the liquid in the channel causes a wave in it. This wave forms pressure nodes—a unique acoustic field inside a channel. A particle takes a position in this field due to its size and density/compressibility. The technique allows manipulation of particles ranging in size from a few nanometres to hundreds of micrometres [232, 233]. There are different modifications, known as standing surface (SSAW) [234, 235], bulk (BAW) [236], and travelling surface (TSAW) [237] acoustic waves. The scheme of acoustophoresis for SSAW (as an example) is presented in Fig. 11A [238, 239].

Acoustophoresis does not require labelling, has a negligible effect on cell viability, and is suitable for particle sorting in the array with high throughput, as described in [240] (see Fig. 11B).

### Microdroplet sorting

The last step is to separate the target droplets from the array. Recognition and sorting speed are important in our case—a higher number of mutants/droplets can be screened since cells continue proliferation during the process. Mutants in those droplets can achieve higher biomass concentration, causing false-positive separation. That is why a fast selection of desired droplets from the array is very desirable. Some possibilities for such types of solutions were described above. However, there are methods that allow the manipulation of single droplets or particles in microfluidic channels, too.

To date, all methods of particle separation in microfluidics are classified as passive and active. As mentioned above, passive methods rely on inertial forces in microchip channels. Active methods require the application of an external field. External forces or fields may include, for example, temperature or concentration gradient, electrical and/or magnetic field, acoustic waves, optical beams, or pneumatic flow control, etc. [220, 241–244]. Some of them are applicable to the separation of objects, like cells, and others are suitable for droplet separation. Here we will consider the methods suitable for the separation of droplets with the size of tens of microns.

#### Hydrodynamic (pressure-controlled) method

**Figure 12.**
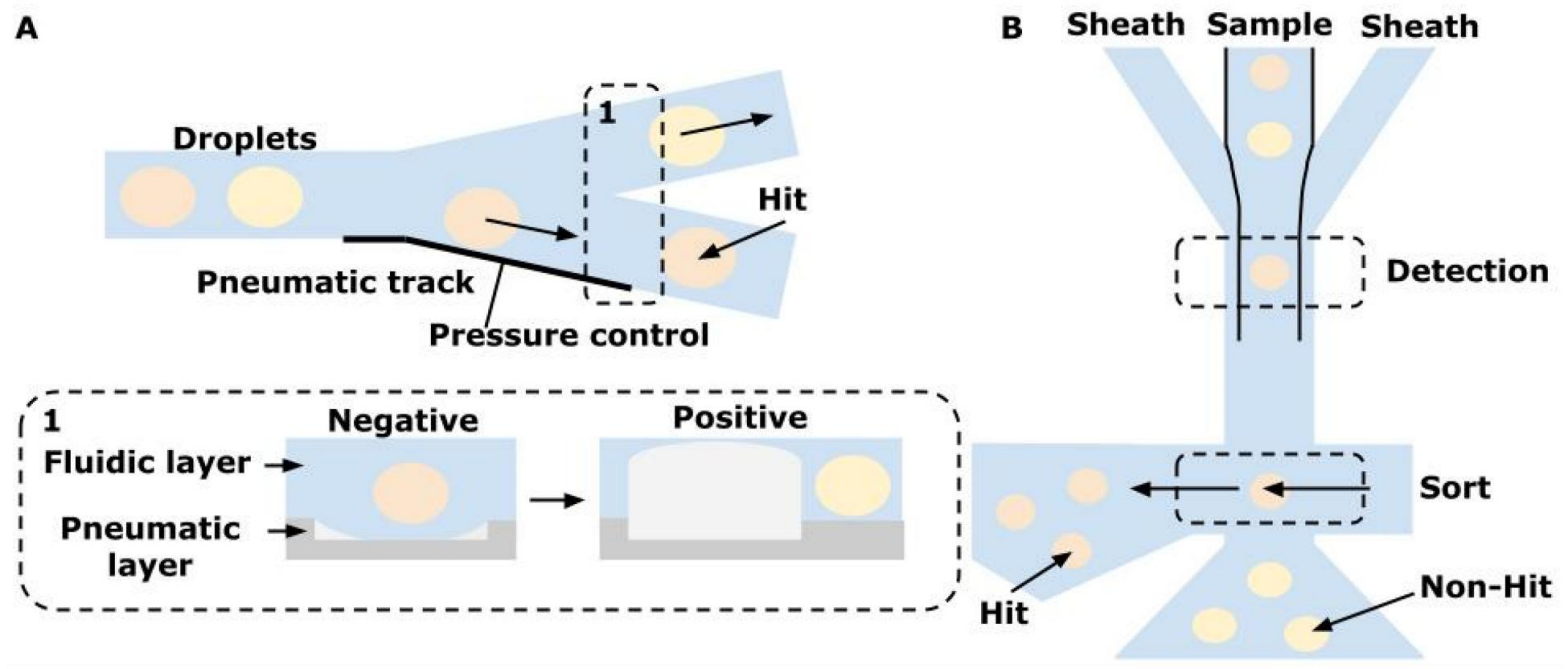
Pressure-controlled methods for droplet separation (images adapted from [220, 247]): (A) droplet sorting is assisted by controlling pressure: negative pressure in the valve causes expansion of the channel and ‘hit’ droplet to continue to flow through the lower microchannel. Positive pressure causes collapse and blocks the lower microchannel, causing droplet flow through the upper microchannel; (B) sorting is performed by pressurized air that deflects ‘hit’ droplets from the initial path to the collection reservoir on-chip that can carry liquids such as culture medium, oil, buffers, etc.

The method is based on controlling the flow of liquid in the channel by changing the external pressure. The most common are two schemes approaches: (i) flexible membrane that simply closes or opens cross-section of the channel [245, 246] also called valves with ‘positive’ or ‘negative’ pressure (see Fig. 12A) [220] or (ii) deflection of the target particle by an additional external flow of liquid—the so-called ‘on-chip sorting’ because of the presence of a collecting camera on a chip (see Fig. 12B) [168, 247]. The advantage of this method is the absence of any influence on the viability of cells and the independence from the size of the sorted particles. The size of the particles that can be sorted using this method could be hundreds of microns but with a low frequency [232, 248]: maximum sorting speed reached ∼2 Hz for 800 µm particles. The expected sorting rate using this method will be approximately in kHz for 40 µm droplets.

#### Dielectrophoresis (DEP)

**Figure 13.**
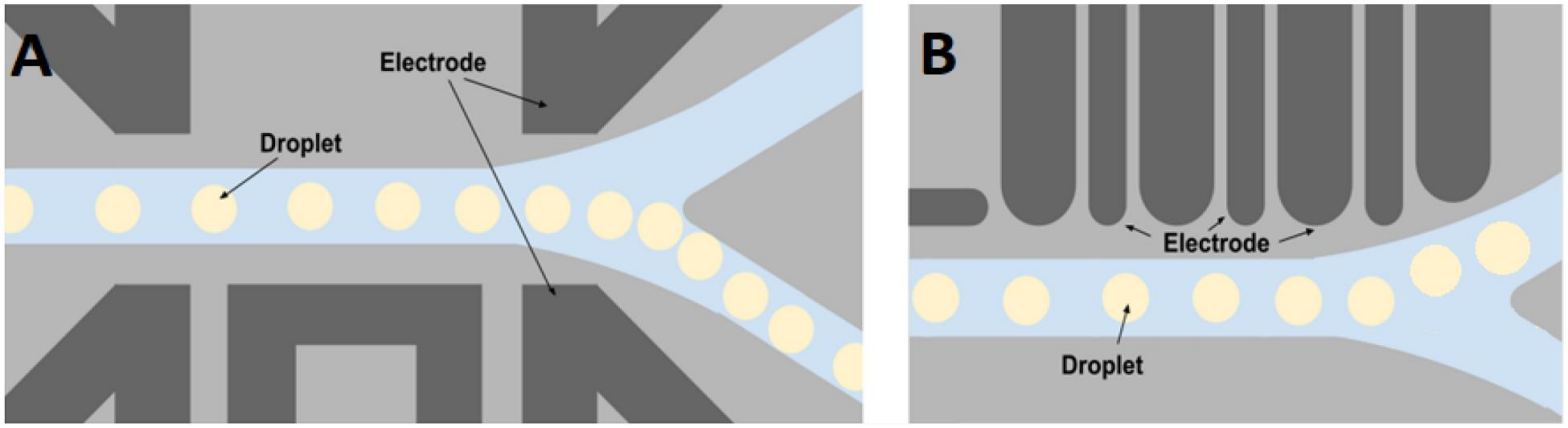
DEP method and several configurations of electrodes (image adapted from [254]): (A) one of the possible configurations of electrodes for droplet sorting; (B) configuration of the periodical structure of switching on–off positive and grounded electrodes when the applied electromagnetic field has a much lower intensity. When applied on a long droplet path, one can reach the desired displacement.

Forces that cause particle/droplet movement appear in a non-uniform electric field due to their polarization. Reviews [249–253] present basics and recent progress in this field. The method does not require external labelling. It is suitable for sorting large w/o droplets [254]. However, the method must be used with precautions: the electromagnetic field can have a negative effect on the viability of cells, as mentioned before and must be adjusted to prevent splitting droplets. The latter effect is also used to control their size [255, 256]. There are different high-throughput sorting device modifications [254, 257–259], where the mentioned negative effects were minimized (see Fig. 13). The authors [254] were able to establish the dependence of the sorting efficiency from droplet’s size for this type of device: the product of multiplication of the droplet volume on the sorting frequency is constant and equals to about 400 000. Based on this, the expected sorting speed will be approximately 5–6 kHz in our experiments.

**Figure 14.**
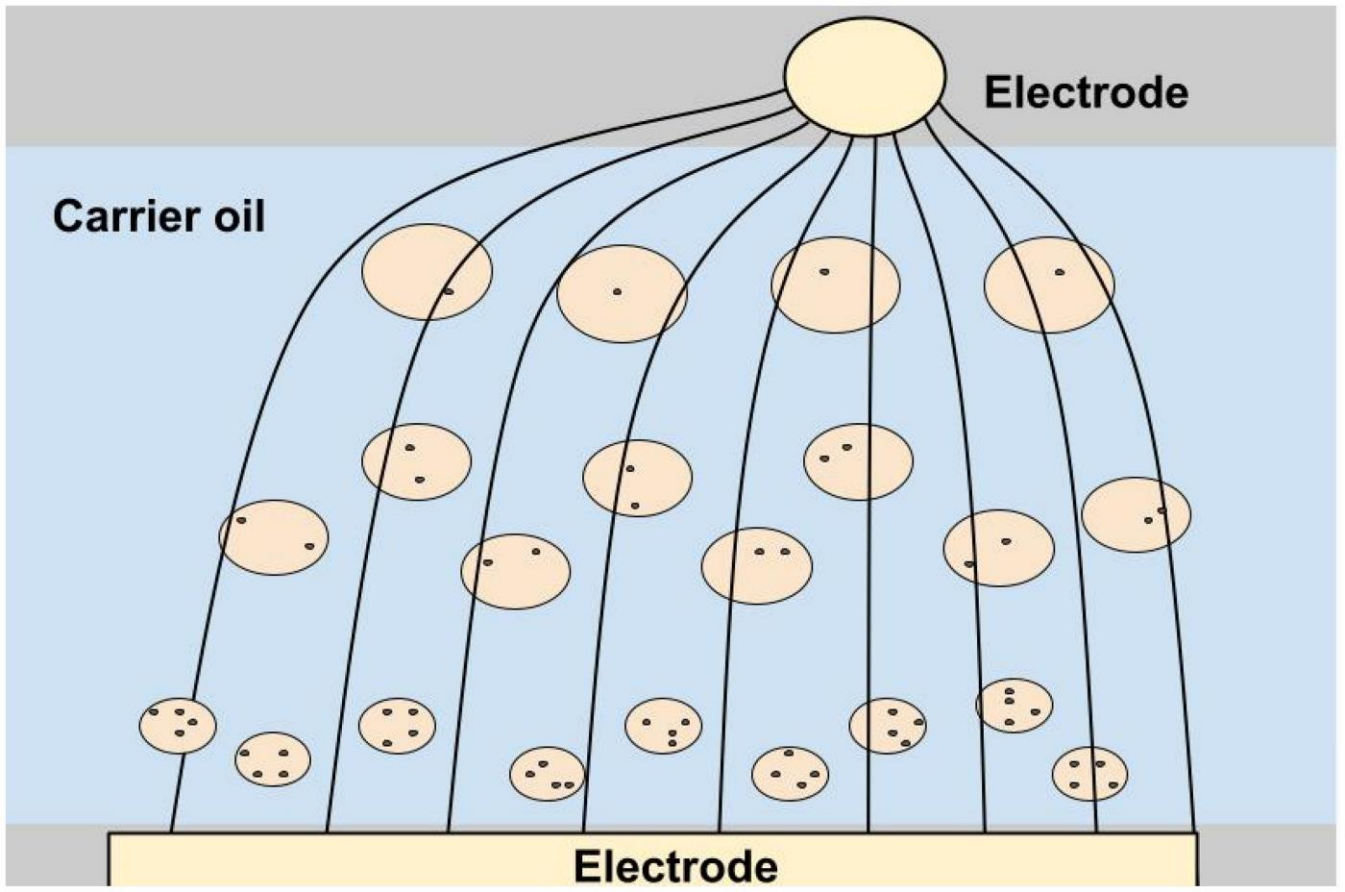
DEP-based sorter scheme: asymmetric electrodes cause a non-uniform electromagnetic field. Under this field, droplets move accordingly to the conductivity of internal media (changes in field in the presence of droplets are not shown). Droplets with higher conductivity move close to the cylindrical electrodes with higher tension of electric field, while droplets with lower conductivity—to the flat electrodes.

It is worth mentioning the appliance of a non-uniform electric field to sort out a considerable number of cells, as proposed in ref. [251] and presented in the case of droplets in Fig. 14. Droplets, in the same way as cells, can be characterized by different electrical conductivity, which depends on cell numbers inside and their activity after incubation. This technique has drawbacks—high deformability (and therefore the possibility of merging/break up) of bigger droplets and the large number of them near cylindrical electrodes, which may cause a shielding effect and bring difficulties during sorting.

#### Acoustophoresis

This method was described in the previous chapter. Here we mention the version of TSAW application: the so-called ‘acoustic valve’—an acoustic wave creates an impulse in the liquid that deflects the particle from its initial trajectory [260].

## Microfluidic system configurations for accelerated selection of mutants

Here, we will briefly represent and discuss several already published microfluidic system configurations (with methods of detection and separation) used for the selection and screening of microorganisms inside droplets. Table 2 presents some papers devoted to similar experiments.

**Table 2.** Microfluidic system configuration for accelerated microorganism selection.

Microorganism	Microdroplet generation/cultivation conditions	Detection and separation	Ref.
Yeast ST4058	Microdroplet size—35 µm (22 pl); oil—HFE-7500; surfactant—EA (1.0%); Incubation: in a syringe for 7 hours	Optical observation, co-cultivation with gene-modified fluorescent *E. coli*; FADS, electric filed sorter	[112]

Cyanobacteria *Synechocystis*	Microdroplet size—50 µm (65 pl); oil—HFE-7500 (3M), surfactant—PicoSurf2 (2.5%); flow focusing, PDMS; cell-density (cells/ml)—1.8*10^6^; Incubation: in syringe for 4 days, 30°C; 80 μmol photons m^–2^*s^–1^	LIF, chlorophyll-associated fluorescence. FADS, electric field sorting system	[113]
		
Algae *C. reinhardtii*	Microdroplet size—40 µm (33.5 pl); oil—HFE-7501, surfactant—PicoSurf1 (3.0%); flow-focusing channel, PDMS;		

Yeast *Y. lipolytica*	Microdroplet size—34 µm (20.6 pl); oil—Novec7500, surfactant—KryJeffD900 (2.5%); cell density (cells/ml)—3.48*10^8^; microdroplet generation productivity (cells/s)—3000; Incubation: in glass vial at 28°C, 1 day	Pico-injection of fluorogenic substrate, LIF electric field sorting system	[115]

Algae *C. vulgari, Ch. reinhardtii,*	Microdroplet size—80 µm (268 pl); oil—FC-40, surfactant—EA surfactant (2%); flow focusing, PDMS; cell density (cells/ml)—3.5*10^6^ Microdroplet generation productivity (cells/s)—60; Incubation: on-chip, 10 days	Image-based processing and observation of biomass growth	[116]

Bacteria *E. coli*	Microdroplet size—20 µm (4.2 pl); oil—HFE-7500 (3M), fluorosurfactant-008 (1.0%); Incubation: 0.5 days	Optical observation, co-cultivation with *E. coli*; FADS	[121]

Algae *E. gracilis*	Microdroplet size—45–95 µm (48–449 pl);	Inertial passive sorter	[158]

Yeast *S. cerevisiae*	Microdroplet size—30 µm (14 pl); oil—HFE-7500, EA-surfactant (0.5%); flow focusing, PDMS; cell density (cells/ml)—2*10^6^	Optical and LIF observations, DLD passive sorter	[172]

Cyanobacteria *Synechocystis* sp. PCC 6803	Microdroplet size—90 µm (380 pl); oil—HFE-7500, Picosurf-1 (2.50%); flow focusing, PDMS; cell density (cells/ml)—5.24*10^6^; Incubation: on-chip, 4 days	Inverted microscope observation with image processing, LIF-detection of chlorophyll fluorescence	[196]

Yeast *Y. lipolytica*	Microdroplet size—30 µm (14 pl); microdroplet generation productivity (cells/s)—2000; Incubation: 36 h, 30°C	Co-cultivation with *E. coli*, FADS; optical inspection and electric field sorting system	[199]

Yeast *P. rhodozyma*	Microdroplet size—30 µm (14 pl); mineral oil; Span80 (2.5%); T-junction, PDMS; cell density (cells/ml)—1.5*10^6^; Incubation: in 2 ml tubes, 40 h, 30°C	Image-based processing sorter with pneumatic control	[261]

Gut anaerobes	Microdroplet size—65–115 µm (143–796 pl); flow focusing; low cell density—2–12% single-cell droplets	Image-based processing sorter with an electric field sorting system	[262]

Here, we will focus on discussing concepts that could lead to new, more efficient chip designs. One consideration is the increase in the number of microdroplets that need to be screened in a short period of time, as we believe that this is the limiting factor—as was shown above, this number must be huge for the successful selection of a few new mutants. Each single cell must be encapsulated with high precision in an individual microreactor using a single microchannel. Therefore, the only feasible way to increase the number of microreactors is the use of several parallel devices (or parallel microchannels on one chip). It is obvious that the hydrodynamic resistance of such a system will be high and limited both by the parameters of the pump and the permissible load on the chip. From the analysis of the published papers (see Table 2), the chip’s design consists of one channel for the generation of droplets with a low initial concentration of the cell culture. Increasing the number of pumps requires more investment. Nevertheless, there are projects that try to make microfluidics less expensive and simply available for users [263, 264]. This is the first limitation factor on the way to increasing the number of microreactors in one experiment. As for the accuracy of this process—each channel can be supplied by passive or active control methods of droplet generation. In this case, encapsulation becomes a stand-alone process on a chip that has all the characteristics of a complete microfluidic system—generation of microdroplets, detection, and selection. On the other hand, such a complex construction has benefits, like significantly reducing the number of microdroplets for later screening by removing unnecessary droplets (including those contaminated by other species). It is interesting to construct a microfluidic system in such a way that the same chip can be used for two purposes. First, an integrated detection/separation system could be used to increase droplet generation precision prior to incubation. Secondly, the same chip is used to select droplets with the highest number of cells (highest biomass content). Such a system must be able to sort droplets using several parameters.

The next steps are recognition and selection. At this stage, the variability of the system increases significantly—the number, size, and content of droplets after incubation vary so widely that it becomes difficult to build a device that can sort droplets by a single parameter and, at the same time, be resistant to noise in the system. Optical observation with image processing software or laser-induced fluorescence and droplet sorting based on this (LIF, FADS) are, therefore, still the most common methods for control at this stage (see Table 2). An often-used method of separation of a single drop is an electric field. Optical methods, however, are characterized by low throughput: in the camera–controller–sorter link, the slowest and most expensive element remains the camera since electric sorters are capable of operating at MGz frequencies. Although the operating frequency of today’s fastest SCARF camera in the world is an impressive 156 TGz [265], the cost of an ‘entry’ level high-speed camera is around 60–80 thousand USD (Phantom TMX 5010, 50720 fps at 1280 × 800) [266].

A MFC for high-throughput droplet selection can be based on passive methods such as DLD, DEP, or SSAW separation. Such configurations seem to be most suitable for sorting an array of droplets, albeit at the cost of lower accuracy. Further droplet selection steps can include optical (with image processing and/or ML, AI-assisted software) or FADS as described above. In this step, several parallel microchannels are also preferred to increase throughput.

Combinations of several microfluidics methods are known as hybrid methods. Some of them (e.g. DLD and electric field) are described in [267–270]. An interesting alternative is the application of ML to control external fields (acoustic or electric)—in this case, manipulation of particles can be achieved without prior information about the shape of the field [271–273]. A recent collection of 36 articles devoted to AI in microfluidics shows growing interest in the field [274]. Automated image processing can help achieve greater accuracy and performance in droplet microfluidics [275]. In our opinion, hybrid systems are the most promising in future experiments on the accelerated selection of various microorganisms, including yeasts.

As for increasing the accuracy and selectivity and expanding the limits of already existing microfluidic technologies for accelerated mutant screening, some methods should also be considered. For example, for the search for new, more effective chromophores, new materials and solutions for easier integration of active components with chips, as well as reducing the cost of microchip manufacturing technology. Microscopic methods are present at every stage of microfluidics, and it is not possible to avoid them completely. So, increasing the availability of high-speed cameras in combination with ML/AI will also make this technology more accessible and efficient.

Also, we would like to mention cell-line contamination, which is not a trivial problem and brings significant experimental errors in biology and medicine [276] and is costly [277]. It could not be avoided completely in microfluidics, too. One of the viable solutions to avoid cross-contamination is using a single-use chip [278] and reducing its cost is preferable in such a case. If yeast is used, various antibiotics in the water-based medium can be applied, but if droplet incubation and sorting take prolonged periods of time, antibiotic potency wears off.

Contamination of cell culture in droplet microfluidics could be prevented before droplet generation—as it was mentioned above, in addition to microscopy, the EIS method could be used for single-cell screening, based on its unique properties [279]. This method requires the creation of specific libraries for microorganism screening and has some disadvantages as described earlier. Also, it is possible that droplets will be contaminated despite efforts made, and they can include unnecessary cells after cultivation. Droplets must be screened after incubation. So, if unnecessary cell morphology differs significantly from the used strain, it could be revealed microscopically, and the use of AI-assisted image processing can help reduce time to achieve purity of cell line.

## Conclusions

It was found that using permeable to oxygen water-in-fluorinated-oil emulsion with stabilizers seems to be most promising both for *Y. lipolytica* and *P. rhodozyma* cultivation conditions using microfluidics. The droplet size suitable for both microorganism growth is in the range of ∼34 μm. Available data indicate that EMS mutagenesis is a promising route for creating mutants considering mutation rates and methods compatibility to droplet generation.

Different techniques and methods suitable for accelerated selection of mutants after incubation in microdroplets were discussed. Methods were chosen considering specific biomarkers of microorganisms (astaxanthin and lipase) and the expected growth of cells inside droplets. Based on literature review, other changes in droplet’s characteristics after incubation were discussed as well—size, mass, optical density, concentration of feeding media, and electrical conductivity.

Possible conceptual designs of MFCs and future perspectives of accelerated selection of yeast mutants based on microdroplets were discussed. We found possibilities to apply sorting techniques known as DLD, DEP, and SSAW to the array of droplets after incubation. They allow one to compose high-throughput microfluidic devices for detection and selection of mutants. Those hybrid methods are more complex but have high throughput and can include LIF, FADS, and AI-assisted image processing. They are the most promising technologies for accelerated selection of microorganisms, including yeasts.
